# Hints Towards the Investigation of the Nature, Cause, and Cure of the Rabies Canina: Addressed to Dr. Haygarth

**Published:** 1789

**Authors:** Thomas Percival

**Affiliations:** Fellow of the Royal Societies of London and Edinburgh, of the Royal Medical Society at Paris, of the Royal Society of Agriculture at Lyons, and of the Philosophical Society at Philadelphia, &c.


VI. *Hints towards the Inveſtigation of the Nature, Cauſe, and Cure of the Rabies Canina: addreſſed to Dr.* Haygarth. *By* Thomas Percival, *M. D. Fellow of the Royal Societies of London and Edinburgh, of the Royal Medical Society at Paris, of the Royal Society of Agriculture at Lyons, and of the Philoſophical Society at Philadelphia,* &c.

YOUR propoſal, my dear friend, for obviating the baneful effects of the bite of a mad dog, communicated November 17, 1788, claimed my immediate attention; and I wrote to you by the ſubſequent poſt, that no delay might take place in the execution of your benevolent volent project *. I have fince read and thought much on the fubject; and fhall now tranfmit to you the refult of my better information, and more deliberate reflection. To your candour I can lay myfelf open without referve; and from your judgment I fhall be equally happy to receive either the correction or confirmation of the following fuggeftions, relative, I. to the nature and caufe; II. to the prevention; and III. to the cure of hydrophobia.

The

* *Copy of Dr.* Haygarth's *Propofal for obviating the Effects of the Bite of a mad Dog.*

Near Wrexham, in North Wales, three men died of canine madnefs in October, 1788.

Thefe melancholy cafes fpread a general alarm. But it ought to give great comfort and fatisfaction to any one who may be bitten, to know that there is a fafe, eafy, and effectual method of preventing infection; which can feldom give pain, or require fkill, and is in the power of every perfon to employ. It is univerfally allowed by phyficians, that the fpittle of a mad animal, infufed into a wound, is the only caufe hitherto known, that can communicate canine madnefs to the human body. This poifon does no immediate mifchief, but is flowly abforbed into the blood; and fufficient opportunity is given to remove it, before any danger can arife. Whenever any perfon is bitten, the plain and obvious means of preventing any future injury, is, firft, to wipe off the fpittle with a dry cloth, and then to wafh the wound with cold

I. I do not perceive any ſtrict analogy between the action of the canine virus and that of the lues venerea, of the ſmall-pox, or of the viper. Theſe evidently affect the lymphatic ſyſtem; and their progreſs into the courſe of circulation cold water; not ſlightly and ſuperficially, but abundantly, and with the moſt perſevering attention; in bad caſes, for ſeveral hours. After a plentiful affuſion of cold water, but not ſooner, warm water may be employed with ſafety and advantage: A continued ſtream of it, poured from the ſpout of a tea-pot or tea-kettle, held up at a conſiderable diſtance, is peculiarly well adapted to the purpoſe. If the canine poiſon infuſed into a wound were of a peculiar colour, as black, like ink, we ſhould all be aware that plenty of water and patient diligence would effectually waſh out the dark die; but this could not be expected by a ſlight and ſuperficial ablution. After a bite has been carefully waſhed, colour it with ſaliva, tinged by ink, &c. When ſome hours have elapſed, waſh out the ſtain. A viſible proof may thus be obtained, how ſoon and perfectly water can cleanſe a wound from ſaliva. As a proof that ſlight waſhing of the wound is not ſufficient to cleanſe it effectually from the poiſon, we may mention, that, in ſome caſes, after inoculation for the ſmall-pox, the poiſonous matter has been attempted to be waſhed out of the wound, by perſons who wiſhed to prevent its effects: yet the inoculated ſmall-pox appeared at its proper period. Theſe unſucceſsful attempts were performed ſecretly, haſtily, and timidly, by a female hand. But in a caſe where the inoculated inciſions were probably waſhed with greater circulation may be readily traced, which is not the caſe with the poiſon of a mad dog. Are we then fundamentally right in the idea, that the bite of a rabid animal operates by abſorption? Might not its effects be, at leaſt as well if not better, explained by aſcribing them to local care, infection was prevented. Such facts teach us the importance of patient perſeverance in waſhing away the poiſon; but they need not abate our confidence that ſuch perſeverance will certainly be ſucceſsful.

The ablution ſhould be accompliſhed with great diligence and without delay; and may be performed by the patient or any aſſiſtant. However, as the apprehenſion of this dreadful diſorder always excites the greateſt anxiety, a ſurgeon's advice and aſſiſtance ought to be obtained, as ſoon as poſſible, in all caſes where the ſkin is injured. He will execute theſe directions moſt dexterouſly and completely. In a bad wound, the poiſon may be conveyed deep into the fleſh, by long teeth or lacerations. In ſuch circumſtances he ſhould open and waſh every ſuſpicious place. And, whenever any painful uncertainty can remain, he ſhould cup and ſyringe. If the bite has been neglected till the inflammation has begun, he ſhould, after ſhaving off the inflamed ſurface, cup, ſyringe, and waſh with double diligence. By this method of purification, it cannot be doubted that every particle of poiſon, and, conſequently, that every cauſe of danger may be effectually removed.

[N. B. Let this paper be paſted up in ſome public places, and in the houſes of ſeveral ſenſible and humane perſons in each pariſh.]

nervous nervous irritation; propagated in different periods of time, according to the varying circumſtances of ſenſibility and irritability, to the brain, and from thence to the fauces, gullet, and ſtomach? Are not all the ſymptoms induced of the nervous and ſpaſmodic claſs? Or, do any marks appear, in the human kind, of a ſpecific vitiation of the fluids?

There ſeems to be a ſtriking reſemblance, in many particulars, between ſome ſpecies of tetanus and the rabies canina*. Now, tetanus is known to be produced, in certain ſtates of the body, by local irritation, without the leaſt ſuſpicion of any abſorption of poiſon, or contamination of the fluids.

Mental

* Dr. Currie, of Liverpool, informs me, that in a caſe of this diſeaſe (tetanus) in which wine proved an effectual remedy, it could only be ſwallowed at certain moments of diminiſhed conſtriction. At other times, if it was ſhewn to the ſufferer, the ſight produced evident diſtreſs; and if it was advanced towards his mouth, it never failed to bring on convulſion. The proper ſeaſon for adminiſtering it was learnt from the patient's ſignal, as he ſpoke with great difficulty: and with every precaution, deglutition was interrupted twice out of three times, that it was attempted, by the acceſſion of convulſion. The wound, which gave riſe to the diſeaſe, was ſo ſlight and ſo nearly healed, that it had eſcaped the patient's notice.

Mental impreſſions have repeatedly excited hydrophobia. Some time ago, I attended a clergyman, who laboured under many of the ſymptoms of it, through the ſhock occaſioned by an official viſit, to one of his pariſhioners, dying of that diſeaſe. He had no opinion of the Ormſkirk powder, but the Tonquin remedy (muſk, cinnabar, &c.) perfectly cured him. As Phyſician Extraordinary to our Infirmary, my advice is ſometimes called for on particular occaſions. I was not long ſince conſulted about a man, bitten by a ſuppoſed mad dog. He had notice. If it had eſcaped the notice of the attendants alſo, and if they had been unacquainted with the actual appearance of tetanus, my very judicious friend conceives it poſſible, that the diſeaſe might have been named hydrophobia.

The following narrative, which is of a ſimilar kind, I received from Dr. Darwin. A young man had his ankle much torn and bruiſed by a fall from two horſes, which he rode at the ſame time, ſtanding upright, on their backs. In a few days, a difficulty of deglutition occurred, and he became totally convulſed, on attempting to ſwallow fluids. Two open ulcers, near the ankle, were then laid into one: the operation however was in vain. Amputation was then propoſed, but rejected: and in ſpite of the uſe of much opium, and mercurial friction, about the fauces, he died on the ſucceeding day. In this caſe, the doctor juſtly obſerves, the hydrophobia was evidently produced by ſympathy with the wounded parts.

the the ufual affections of the difeafe, though in a flight degree, which were removed by mercurials and antifpafmodics. When the man was recovered, and qualified to ftate minutely, and without anxiety, the circumftances antecedent to his attack; I was perfectly convinced that his malady had originated folely from the terrors of imagination. In fuch cafes, there can affuredly be no ground to impute the malady to the abforption of any poifon; and it muft be afcribed entirely to nervous irritation.

The accurate Morgagni has related a cafe of hydrophobia, occafioned by the bite of an enraged cat, which was not mad. The virus in this cafe, if virus is to be fuppofed, could not be of the fpecific kind belonging to an hydrophobic animal. And the fymptoms are to be accounted for in the fame way, in which we explain the confequences of a wounded tendon, or the fplinter of a fractured bone, when fuch caufes produce a locked jaw or tetanus.

The virus of the fmall-pox, or of the lues venerea very rarely fails of producing its baneful effects on the body, when applied. Whereas the bite of a mad animal is not found to be deleterious in a very large, though indeterminate number of cafes. Is not fuch a difference thus explicable?

explicable? The former is tranſmitted into the ſyſtem by abſorption, through a ſeries of veſſels, which are uniform and regular in their action. The latter is, perhaps, poiſonous only to certain nerves, under certain conditions; ſo that the chance is always great againſt its operation. Juſt ſo it is with wounds or injuries of the tendons: Theſe are very rarely ſucceeded by a locked jaw, and only under peculiar circumſtances.

That a nerve is capable of irritation, independently of the brain, whilſt the vital energy ſubſiſts, is evinced by the contractions of the heart, procuced by pricking or wounding it, when taken out of the body. May not this be the power which is firſt excited by the canine poiſon; and which requires an indefinite time to operate, before it communicates with the brain, or rouſes the perception of injury done to the ſyſtem? We are informed by a celebrated anatomiſt (Monro), that the nerves reſemble the brain in ſtructure; and that, as they proceed in their courſe, they acquire additional energy.

Morgagni aſſerts, that the poiſon of a mad animal has been known to remain latent even for twenty years, till being excited into action by ſome cauſe, certain deſtruction was the con-

ſequence.

ſequence. This information, however, he delivers, not on his own authority, but as what he believes to be founded in truth. Whatever doubt may be entertained of its credibility, there can be none of the caſe, which the ſame author relates, of a boy under his own inſpection, in whom the ſymptoms of the hydrophobia came on five months after a bite in the leg, by an animal not then known to be mad. And Dr. Vaughan has given us lately the hiſtory of a patient, who was bitten in September, without any appearance of canine infection till the ſixth of June following. It is evident, therefore, that the injury done may long remain topical, and ſo ſlight as not to be perceived. This occurs in affections confeſſedly originating in the nerves*.

A difficulty in ſwallowing liquids, or the dread of water, is not always a concomitant of

* Some time ſince, I attended a lady, who had received a bruiſe on the os ſacrum, by a fall, when ſhe was young. She ſoon recovered from its effects: but eighteen years afterwards, the rheumatiſm fixed on the part, was attended with unuſually excruciating pain, and long reſiſted the remedies, commonly employed with much more ſpeedy ſucceſs in that diſorder. In this caſe, we may preſume, there ſubſiſted a morbid topical affection of the nerves, which a ſubſequent cauſe rendered manifeſt.

the the other ſymptoms, incident to perſons bitten by mad animals. This only ſhews a variation in different ſubjects, in the ſympathetic powers of the nervous ſyſtem. And I have lately met with an equal degree of diverſity in an affection of the jaw, produced by an injury from a bodkin in the tendons of the hand. The wound ſoon healed without any inflammation remaining; the pain was not conſtant, nor at any time intenſe. No ſtiffneſs was felt in the jaw; but, on the contrary, a weakneſs and inability to prevent its dropping. The lady chewed and ſwallowed with eaſe; but on reading aloud, ſhe had a pain in the maxillary articulation: nor could ſhe prevent frequent yawning. This patient had been long an invalid, previous to the accident; and there was reaſon to ſuſpect, in her caſe, ſome antecedent morbid affection both of the brain and of the ſpinal marrow..

Diſſections have hitherto thrown little or no light on the nature of the rabies canina. Morgagni, though he has related many hiſtories, declines to draw any concluſions from them; obſerving from a compariſon of all, that the dead differ much more from each other than the living. We muſt therefore be content with conjecture; and adopt with diffidence that hypothesis, which appears most consonant to reason and to truth. And this must be our clue in the treatment of persons who may be, or who are actually sufferers by the bite of rabid animals: for we have yet to learn that mode of practice, on the success of which we can place any just dependence. Your proposal, therefore, of a more likely mean of prevention than any yet suggested, merits the cordial thanks of the public.

II. Agreeably to the rules of your printed paper, let the patient, with as much expedition as possible, wipe off the spittle of the dog with a dry cloth, (suppose his handkerchief, as being always at hand) and then, abundantly and with the most persevering attention, wash the wound with water. The preference you give to cold water, for the first ablution, is judicious; and accords with the idea, above advanced, that the nerves are the parts alone injured by the canine virus. They may thus perhaps be rendered torpid, and the virus may be greatly diluted or washed away, before they recover such sensibility as to be capable of suffering from its action. Ligatures, placed above and below the wound, would contribute to the benumbing action of the cold water*. When this has been sufficiently applied, warm water should be used, not only as a better solvent, but to produce a flow of blood; which, coming from numberless small vessels, may tend to complete the cleansing of the wound †. At this time the cupping-glass would be a good auxiliary ‡.

When the part bitten may be supposed to be as much freed from the poison as can be accomplished by ablution, additional security may, perhaps, be afforded by bathing it very well with the gastric juice of a healthy animal, recently killed. The penetrating quality of this fluid; its energy as an almost universal solvent; and its power of rendering even poisons not only innocent, but nutritious, promise a salutary operation under the circumstances described. If we are to expect an antidote to the canine virus, this fluid seems more likely to prove such, than any other substance yet discovered. For I have

* From the experiments of Abbé Fontana, concerning the bite of the viper, it appears, that ligatures, round the bitten limb, had a very salutary effect.

†. Plunging the part, infected with the poison of a viper, into warm water, and keeping it therein some time, appeared to Abbé Fontana to be truly advantageous.

‡. Vide Celf. lib. VII. cap. 23. § 3.

some

ſomewhere ſeen it recorded as a fact, that a piece of meat, imbued with the ſaliva of a mad dog, has been ſwallowed by another dog with impunity. The gaſtric juice of a carnivorous is more active than that of a graminivorous animal. A cat, a dog, or carrion crow may therefore be killed for the purpoſe. From the laſt, Abbé Spallanzani obtained this fluid in great purity without injury to the bird, by introducing bits of dry ſponge into the ſtomach.

If the gaſtric juice be not attainable, probably the ſaliva of a healthy young perſon would be the beſt ſubſtitute; and it might be procured by the chewing of rennet, which has been well freed from the ſalt *. We are informed by Celſus, that the Pſylli ſucked, without injury, the poiſon infuſed by ſerpents. The ſpittle is demulcent, inviſcating, and capable of chang-

* The rennet cannot be entirely freed from the ſalt, with which it is preſerved, without depriving it of a conſiderable portion of gaſtric juice. Perhaps the ſalt itſelf may have ſome ſalutary powers. Abbé Raynal aſſerts, that it counteracts the poiſon of the American Manchineel tree, vol. V. p. 369. Celſus recommends it in the bite of a viper, lib. V. 27. In Virginia, when the Indians are bitten by a rattle ſnake, they lay to the part affected the radix Senekæ, well chewed, by which it muſt be fully impregnated with ſaliva. See Mead's Works, p. 44.

ing the qualities of bodies by its fermentative nature. One or other of theſe applications muſt therefore be renewed ſeveral times daily; and the part ſhould be afterwards covered with a cataplaſm.

In recommending the ſeveral foregoing means of prevention, I would not be underſtood to preclude exciſion of the part bitten, when the patient has courage to undergo the operation, and it can be accompliſhed without danger. But much time may be loſt, before the ſurgeon arrives; the ſufferer may long reſiſt all ſolicitations to ſubmit to the knife; the wound may have been inflicted on the face, or near ſome large blood veſſel; or there may be ſo little probability of the madneſs of the dog, as to render it unjuſtifiable to ſubject the patient to preſent pain, and future deformity. In all theſe caſes, your plan of ablution promiſes much benefit; is liable to no objections; may be inſtantly executed; and will allow ſufficient leiſure for a careful and complete exciſion. The application of cauſtics has been found unſucceſsful: and the exploſion of gun-powder could hardly take place in a bleeding wound: nor could we ſecure its extenſion through the oblique courſe of the animal's teeth. To this laſt conſideration you have judiciouſly paid attention, by directing water to be poured on the wound from the ſpout of a tea-kettle.

On the ſuppoſition that the hydrophobia is a diſeaſe of the nervous claſs, the means to obviate its acceſs ſhould ſeem to be ſuch as diminiſh ſenſibility and irritability, and give tone and vigour to the ſyſtem. Bark, ſteel, cuprum ammoniacum, or flowers of zinc may therefore be adminiſtered; and the patient ſhould be directed to uſe the cold bath frequently. If his mind be agitated, or depreſſed, laudanum will occaſionally be required. I have reaſon to think, that the late Mr. Hill, of Ormſkirk, in ſome caſes, joined opiates with his once celebrated ſpecific. To the uſe of this popular noſtrum, if the patient entertain much confidence in it, there can be no reaſonable objection; becauſe only a few doſes of it are recommended, and conſequently it cannot long interfere with the exhibition of other more active medicines. Perhaps it may be degraded in the eſtimation of the faculty, by not being adminiſtered in adequate quantities, or with ſufficient perſeverance. To infallibility no remedy yet known can have any juſt pretenſions: and the inſtances of the failure of the Ormſkirk powder are not more numerous than thoſe which have been recorded of every other mean hitherto employed. It is indeed compoſed of ingredients of no great efficacy in ordinary caſes, ſo far as we can rely on the analyſis which has been given of it. But activity or inertneſs are relative, not abſolute, qualities in medicinal ſubſtances, and their energies, with reſpect to the human body, depend on the ſtate of the animal ſyſtem at the period when they are adminiſtered. Experience, therefore, is the only teſt of their inefficiency or power. In other caſes we find that apparently ſimple means produce very ſalutary effects. Thus milk is an uſeful ſolvent when arſenic has been received into the ſtomach: and I have been informed, by a gentleman of knowledge and veracity, that, in South Carolina, he has ſeen the poiſon of the rattle ſnake ſpeedily counteracted by the juice of plantane and horehound *. I have enlarged on this topic,

* This gentleman was on a viſit in South Carolina when a ſervant was bitten in the hand by a rattle ſnake. Ligatures were inſtantly applied near the part, but the hand and arm ſwelled; the jaw became ſomewhat locked; and the man appeared to be in a ſtate of conſiderable danger. The ſurgeon adminiſtered to him, by ſpoonfuls, at ſhort intervals, the juice topic, not from any degree of faith in the powers of the Ormſkirk powder as an antidote to the canine poiſon, but to obviate prepoſſeſſions, which miſlead inveſtigation and obſtruct the attainment of a ſucceſsful remedy in this moſt dreadful of human diſorders.

III. The acceſſion of canine madneſs is uncertain as to the diſtance of time from the bite, and juice of plantane and horehound; and the wounded part was covered with a cataplaſm of the ſame herbs bruiſed. The ſalutary effects of theſe remedies were ſoon apparent, and the ſervant afterwards perfectly recovered. Mr. Cateſby is of opinion that no remedy is yet diſcovered for the bite of a rattle ſnake; yet he acknowledges the having ſeen recoveries, but imputes them to the efforts of nature and to the ſlightneſs of the bite. How far this may be true of the caſe above related I ſhall not attempt to decide; but the obſervation reminds me of an important diſtinction, made by Boerhaave, on the comparative danger of infection by a mad dog under the different ſtages of his malady.

Since this paper was written, Dr. Darwin has obligingly communicated to me a paſſage from Abbé Groſier's Deſcription of China, (Vol. I. page 279) in which it is ſaid, "that "people are often bitten by ſerpents at Tang-king, and are "cured by applying to the wound a ſtone reſembling a cheſ- "nut, which is called ſerpent ſtone. The ſtone, after preſ- "ſing out the blood, is applied to the wound, to which it "adheres, ſucking out the poiſon, and then falls off. It is "then and the symptoms by which it first manifests itself. But frequently the cicatrix becomes hard and elevated; pains shoot from it towards the head; it is surrounded with livid or red streaks; and the wound breaks out afresh. The friends of the patient should be apprised of the appearances which are to be suspected, that they may

" then well washed in lime water and dried, and applied a " second time to the wound; or another stone may be used; " and thus the poison is extracted." Whether the Abbé's observations may be relied upon it is not easy to decide; but certain it is that an absorbent earth acts forcibly by the attraction of capillary tubes, as any one may experience by holding a fresh-burnt tobacco pipe between the lips. The poisoned wound, however, must be both recent and superficial to be cleansed by such a remedy. The Bramins of India expose to sale an antidote, which they pretend to be taken out of the head of the hooded serpent. Redi, however, asserts, that it is made of calcined hartshorn, which is an active absorbent. On this occasion you will recollect the fact lately transmitted to you by Dr. Waterhouse, Professor of Physic at Cambridge, in New England, that when a rattle snake bites the nose of a dog, he digs a hole in the ground, lays his head in it, and is commonly cured. It is probable that the surface of the earth is in a very adust, and consequently absorbing state, in those parts of America where such accidents occur; and that the nose of this animal is less susceptible of being injured by poison, from its coldness, and from the slime with which it is covered, than any other part of the body.

be be fedulouſly watchful, and give inſtant notice to the medical practitioner employed of the change which has occurred. In this firſt ſtage of the diſeaſe a large doſe of thebaic extract ſhould be adminiſtered, to obtain, if poſſible, a truce. This remedy ſeems appropriate to various ſymptoms of the hydrophobia; and we might have hoped it would have merited our entire reliance, from Mr. Pott's aſſurances of its great efficacy in the painful mortification of the toes, which is ſomewhat analogous in its commencement. But experience has ſhewn that it produces no laſting benefit; and recourſe muſt be had, without delay, to ſome other medicine, adequate to counteract the impreſſion of the canine poiſon by another impreſſion, equally forcible, on the nervous ſyſtem *. The qualities of the fox glove, and its quick action, ſeem to recommend it to our trial on this occaſion. It affects the brain very powerfully; excites long-continued ſickneſs and vomiting; and has been ſuppoſed to produce a copious flow of

* Probably the efficacy of half drowning the patient, which practice is ſaid to have been ſucceſsful in a few inſtances, muſt have ariſen from the counter-impreſſion made on the nervous ſyſtem.—*Vide* Vanſwieten. Comment. Vol. III. page 559.

 ſaliva.

faliva *. The laft effect may be promoted by mercurial unctions, from which, in a few inftances, fome benefit feems to have been derived.

The propagation of local nervous irritation to the brain is manifeft in certain cafes of epilepfy, and is obviated by tight ligatures. This fact feems to point out a fimilar treatment of the hydrophobous patient, as foon as the part bitten fhews any figns of infection. Whether excifion may then be advifable muft be determined by its fituation, and by circumftances, about which the attending phyfician or furgeon can alone decide. Should the wound open, it may be dilated, and wafhed with tepid water; after which the gaftric juice may be again applied. It has been found highly ferviceable in foul ulcers, and is quick in producing its effects. It fweetens their fœtor; eafes lancinating pains; and corrects even the cancerous acrimony. Over the dreffings a fermenting cataplafm may be laid, compofed of flour, honey, water, and yeaft.

Such are the additions to your means of prevention, and fuch the curative plan, which I

* See Withering on the Fox Glove, page 184.

take the liberty of fuggefting to your confideration. Both may perhaps feem fuperfluous to you, whofe generous zeal, for the good of your fellow creatures, has elevated hope into confidence, and makes you fay of the fufferer, in the language of a well-known amiable character, "he fhall not die." I heartily wifh that experience may fully confirm your fanguine expectations. But though I highly approve of your propofal as judicioufly conceived, bidding fair to be fuccefsful, and fo eafily practicable, that it fhould be adopted, after the bite of every animal, to obviate even unfufpected danger; yet at prefent I can regard it only as a rational hypothefis on a fubject ftill remaining in much obfcurity. We are ignorant of the peculiar properties of the canine virus; of the mode of its communication; and the parts of the animal fyftem affected by it. Are we then qualified to advance one ftep beyond conjecture? and ought we to reft fatisfied with mere ablution, when it is uncertain what time is required for the agency of the deadly poifon, and whether its baneful operation may not be accomplifhed even before the completion of the fpeedy procefs you have pointed out? But admitting, what I fincerely hope may be found to be the truth, that the plentiful affuſion of water will prove an effectual means of prevention, there will ſtill remain the neceſſity of a curative plan in thoſe caſes, wherein the former has been neglected, or in which the diſeaſe has unexpectedly occurred.

I ſhall think myſelf happy if the hints I have propoſed merit your approbation: but it will render me ſtill more happy if they incite you to extend your reſearches, and afford aid to your enlightened mind, in perfecting its diſcoveries on the important object of your inveſtigation. *Fungar vice cotis — non inani munere.*

*Mancheſter,*
May 1, 1789.

### POSTSCRIPT.

Auguſt 11, 1789. — Before this paper was ſent to the preſs I had not peruſed the valuable Obſervations on the Cauſe and Cure of the Tetanus, by Dr. Ruſh, of Philadelphia. He has obligingly ſent me the volume of Medical Inquiries in which they are contained; and in the Appendix to his Eſſay I am much pleaſed to find a coincidence in our ideas of the analogy logy between tetanus and hydrophobia. I ſhall tranſcribe the paſſages which have a reference to it.

" The more I have conſidered the cauſes

" and ſymptoms of hydrophobia, the more I

" am diſpoſed to aſcribe it to the ſame proxi-

" mate cauſe as the tetanus. 1. They both af-

" fect the muſcles of deglutition. I have lately

" ſeen a tetanus brought on by a fractured leg,

" in which an attempt to ſwallow the ſmalleſt

" quantity of liquid produced the ſame ſudden

" and general convulſions which occur in the

" hydrophobia. 2. They both proceed from

" cauſes which appear to be related to each

" other, viz. from wounds, and from the action

" of cold, after the body has been previouſly

" weakened by heat and exerciſe. 3. They

" both ſometimes appear as ſymptoms of the

" ſame idiopathic diſorder, viz. the hyſteria.

" 4. They both yield to the ſame remedies, viz.

" to the excitement of an inflammation in the

" wounded part of the body, or to a long-con-

" tinued diſcharge of matter from it, and to

" mercury.

" If more facts ſhould occur, which ſhall

" ſhew the relation that the tetanus and hydro-

" phobia have to each other, perhaps we may

" be

" be led to conclude, that the wound inflicted

" by the teeth of a dog sometimes acts in the

" same manner in producing hydrophobia that

" wounds made by a nail, or any other lacera-

" ting instrument, act in producing tetanus,

" and that both diseases may be prevented or

" cured, with equal certainty, by the same tonic

" remedies."

The tetanus is ascribed, by Dr. Rush, to relaxation: and in the cure of it, he says, it is necessary not only to restore the ordinary tone of the system, but to produce something like the inflammatory diathesis. A splinter under the nail, he affirms, produces no convulsions, if pain, inflammation, or suppuration follow the accident; and that the spirit of turpentine acts, by the excitement of pain and inflammation, in all wounds and fractures of tendinous parts. He has never known a single instance of tetanus from a wound to which this remedy had been applied in time. In the island of St. Croix the negroes, he relates, always apply a plaster, made of equal parts of salt and tallow, to their fresh wounds, to prevent the locked jaw; and that the salt never fails to produce some degree of inflammation.

These facts confirm the propriety of applying the gastric juice to the part bitten by a supposed rabid animal, because it is powerfully stimulant, as well as penetrating, solvent, and detergent: and if rennet be substituted, as recommended in page 307, there will be no necessity for separating any more of the salt than what adheres to it superficially.

